# Prognostic impact of HER2-low positivity in patients with HR-positive, HER2-negative, node-positive early breast cancer

**DOI:** 10.1038/s41598-023-47033-8

**Published:** 2023-11-11

**Authors:** Shohei Shikata, Takeshi Murata, Masayuki Yoshida, Hiromi Hashiguchi, Yukiko Yoshii, Ayumi Ogawa, Chikashi Watase, Sho Shiino, Hirokazu Sugino, Kenjiro Jimbo, Akiko Maeshima, Eriko Iwamoto, Shin Takayama, Akihiko Suto

**Affiliations:** 1 Department of Breast Surgery, National Cancer Center Hospital, 5-1-1 Tsukiji, Chuo-ku, Tokyo, 104-0045 Japan; 2 Department of Diagnostic Pathology, National Cancer Center Hospital, 5-1-1 Tsukiji, Chuo-ku, Tokyo, 104-0045 Japan

**Keywords:** Breast cancer, Prognostic markers

## Abstract

Adjuvant therapy for patients with hormone receptor (HR)-positive, human epidermal growth factor receptor 2 (HER2)-negative, node-positive, early breast cancer (EBC) remains challenging. The prognostic significance of HER2-low positivity in these patients is not fully understood. In our retrospective study, we analyzed 647 patients with HR-positive, HER2-negative, node-positive EBC, stratifying them into three cohorts based on axillary lymph node involvement, tumor size, and characteristics. Cohort 1 included patients with either ≥ 4 positive axillary lymph nodes or 1–3 positive nodes with histological grade 3 or tumor size ≥ 5 cm. Cohort 2 consisted of patients with 1–3 positive nodes, histological grade < 3, tumor size < 5 cm, and Ki-67 ≥ 20%. Cohort 3 comprised patients with 1–3 positive nodes, histological grade < 3, tumor size < 5 cm, and Ki-67 < 20%. We compared invasive disease-free survival (IDFS) and distant relapse-free survival (DRFS) between HER2-low (IHC1+ or IHC2+/FISH−) and HER2-zero (IHC0) groups in each cohort. In cohort 1, HER2-low patients exhibited significantly better 5-year IDFS (84.2% vs. 73.6%, p = 0.0213) and DRFS (88.2% vs. 79.8%, p = 0.0154). However, no significant differences were observed in cohorts 2 and 3. Our findings suggest HER2-low positivity as a prognostic factor in HR-positive, HER2-negative, and node-positive EBC.

## Introduction

Hormone receptor (HR)-positive/human epidermal growth factor receptor 2 (HER2)-negative is the most common breast cancer subtype; accounting for approximately 70% of all breast cancer^1^. For the longest time, adjuvant systemic therapy for this subtype has only included endocrine therapy and chemotherapy, but recently the efficacy of targeted therapy such as cyclin-dependent kinase 4/6 (CDK4/6) inhibitor has been demonstrated^2^. In the monarchE trial, CDK4/6 inhibitor plus standard endocrine therapy has shown statistically significant improvement in invasive disease-free survival (IDFS) compared with endocrine therapy alone for HR-positive, HER2-negative, node-positive early breast cancer (EBC) with a high risk of recurrence. In that trial, a high risk of recurrence was defined as 4 or more positive axillary lymph nodes (ALNs), or 1–3 positive ALNs, high Ki-67 index (≥ 20%), and either histological grade 3 or tumor size ≥ 5 cm^2^. Additionally, other HER2 targeting therapies such as trastuzumab, pertuzumab, lapatinib, and trastuzumab emtansine (T-DM1) have not been proven to be effective in HR-positive, HER2-negative EBC^3^. However, a novel anti-HER2 therapy such as Trastuzumab deruxtecan (T-DXd), an antibody–drug conjugate targeting HER2, has recently been reported effective not only in HER2-positive but also in HER2-low breast cancer^4, 5^.

HER2 is an important indicator of breast cancer prognosis, and simultaneously an essential target of pharmacotherapy for breast cancer. According to the American Society of Clinical Oncology/College of American Pathologists (ASCO/CAP) 2018 guidelines, HER2 status is defined by immunohistochemistry (IHC) and fluorescence in situ hybridization (FISH). Breast cancer with the IHC3+ or IHC2+/FISH-positive are classified as HER2-positive, and IHC0, IHC1+ or IHC2+/FISH-negative as HER2-negative^6^. Furthermore, the current HER2 evaluation can distinguish HER2-low (IHC1+ or IHC2+/FISH-negative) from HER2-zero (IHC0). In the DESTINY-Breast04 trial, T-DXd showed improvement in progression-free survival and overall survival (OS) of HER2-low metastatic breast cancer^5^. The DESTINY-Breast05 trial is now ongoing to investigate the efficacy of T-DXd as an adjuvant therapy for HER2-positive EBC^7^. These therapeutic developments have focused attention on HER2-low as a new subtype and therapeutic target. However, little is currently known about the clinical characteristics and prognosis of HER2-low patients. In particular, whether patients with HER2-low have different clinicopathological features from patients with HER2-zero remains controversial. In the case of triple-negative, HR-negative/HER2-negative EBC, the prognosis of patients with HER2-low was better than that of patients with HER2-zero^8^. By contrast, no differences in prognosis were observed between HER2-low and HER2-zero in HR-positive, and HER2-negative EBC^9, 10^. In these previous reports on HR-positive, and HER2-negative EBC, the majority of patients were node-negative. It remains unknown whether HER2-low is prognostically useful when patients are node-positive. Furthermore, the prognostic significance of HER2-low in high-risk patients eligible for CDK4/6 inhibitors has not been fully studied. If HER2-low status is shown to be a prognostic factor in HR-positive, HER2-negative, and node-positive EBC, HER2 expression level would be a promising factor in determining treatment strategies.

Accordingly, the primary aim of this study was to investigate whether there are prognostic differences between HER2-low and HER2-zero in HR-positive, HER2-negative, and node-positive EBC. Additionally, we aimed to evaluate the prognostic difference between HER2-low and HER2-zero stratified by risk of recurrence, according to the risk classification used in the monarchE trial.

## Results

### Patient characteristics

A total of 647 HR-positive, HER2-negative, and node-positive breast cancer patients were included in this study. Clinicopathological findings of all patients are shown in Table 1. There were 228 patients (35.3%) and 419 patients (64.7%) in the HER2-zero and HER2-low groups, respectively. Significant differences in histological type, postoperative radiotherapy, and endocrine therapy were observed between the two groups. The frequency of ILC in the HER2-low group was higher than in the HER2-zero group (*p* = 0.043). Postoperative radiotherapy was performed more often in the HER2-zero than in the HER2-low group (*p* = 0.011). Endocrine therapy was administered more frequently in the HER2-low than the HER2-zero group (*p* = 0.027). No significant differences between the two groups were seen for other clinicopathological features. The baseline characteristics were well-balanced in the two groups. The HER2-zero group, contained 124 (54.4%), 34 (14.9%), and 70 (30.7%), in cohorts 1, 2, and 3, respectively. Meanwhile, the HER2-low group, contained 227 (54.2%), 73 (17.4%), and 119 (28.4%) patients in cohorts 1, 2, and 3 respectively.

**Table 1:** Patients’ characteristics (all patients).

Category	HER2-zero (n = 228)		HER2-low (n = 419)		p-value
	N	%	N	%	
Age, years					
< 65	175	(76.8)	325	(77.6)	0.814
≥ 65	53	(23.3)	94	(22.4)	
Sex					
Female	227	(99.6)	415	(99.1)	0.474
Male	1	(0.4)	4	(1.0)	
Menopausal status					
Pre	103	(45.2)	218	(52.0)	0.096
Post	125	(54.8)	201	(48.0)	
ER status					
Negative	2	(0.9)	3	(0.7)	0.823
Positive	226	(99.1)	416	(99.3)	
PR status					
Negative	22	(9.7)	35	(8.4)	0.579
Positive	206	(90.4)	384	(91.7)	
HER2 status					
IHC0	228	(100)	0	(0)	< 0.001
IHC1+	0	(0)	292	(69.7)	
IHC2+/FISH−	0	(0)	127	(30.3)	
Histological type					
IDC	180	(79.0)	354	(84.5)	0.043
ILC	36	(15.8)	36	(8.6)	
IDC + ILC	3	(1.3)	6	(1.4)	
Others	9	(4.0)	23	(5.5)	
HG					
1	32	(14.0)	80	(19.1)	0.254
2	127	(55.7)	215	(51.3)	
3	69	(30.3)	124	(29.6)	
Tumor size, cm					
< 2	78	(34.1)	163	(38.9)	0.196
2–5	107	(46.9)	198	(47.3)	
≥ 5	43	(18.9)	58	(13.8)	
Number of positive lymph nodes					
1–3	157	(68.9)	306	(73.0)	0.261
≥ 4	71	(31.1)	113	(27.0)	
Ki-67 index, %					
< 20	108	(47.4)	187	(44.6)	0.504
≥ 20	120	(52.6)	232	(55.4)	
Breast surgery					
BCS	84	(36.8)	135	(32.2)	0.235
TM	144	(63.2)	284	(67.8)	
Axillary surgery					
SLNB only	17	(7.5)	48	(11.5)	0.106
ALND	211	(92.5)	371	(88.5)	
RT					
Yes	159	(69.7)	250	(58.7)	0.011
No	69	(30.3)	169	(40.3)	
CT					
Neoadjuvant	24	(10.5)	60	(14.3)	0.385
Adjuvant	140	(61.4)	244	(58.2)	
No	64	(28.1)	115	(27.5)	
ET					
Yes	214	(93.9)	408	(97.4)	0.027
No	14	(6.1)	11	(2.6)	
Risk group					
Cohort 1	124	(54.4)	227	(54.2)	0.660
Cohort 2	34	(14.9)	73	(17.4)	
Cohort 3	70	(30.7)	119	(28.4)	

*ER* estrogen receptor, *PR* progesterone receptor, *HER2* human epidermal growth factor receptor 2, *IDC* invasive ductal carcinoma, *ILC* invasive lobular carcinoma, *HG* histological grade, *BCS* breast-conserving surgery, *TM* total mastectomy, *SLNB* sentinel lymph node biopsy, *ALND* axillary lymph node dissection, *RT* radiotherapy, *CT* chemotherapy, *ET* endocrine therapy, *FISH* fluorescence in situ hybridization.

HER2-zero group: Patients with immunohistochemistry (IHC) 0 score.

HER2-low group: Patients with IHC1+ or IHC2+/FISH− scores.

Cohort 1: Patients with ≥ 4 positive axillary lymph nodes (ALNs), or 1–3 positive ALNs, and either histological grade (HG) 3 or tumor size ≥ 5 cm.

Cohort 2: Patients with 1–3 positive ALNs, HG < 3, tumor size < 5 cm, and high Ki-67 index (≥ 20%).

Cohort 3: Patients with 1–3 positive ALNs, HG < 3, tumor size < 5 cm, and low Ki-67 index (< 20%).

Table 2 lists clinicopathological features according to the three cohort groups. There were 351 (54.3%), 107 (16.5%), and 189 (29.2%) patients in cohorts 1, 2, and 3, respectively. In cohort 1, there were 124 patients (35.3%) in the HER2-zero group and 227 patients (64.7%) in the HER2-low group. Postoperative radiotherapy was performed more frequently in the HER2-zero group than in the HER2-low group (*p* = 0.001). Endocrine therapy was administered more often in the HER2-low group than in the HER2-zero group (*p* = 0.009). There were no significant differences between the two groups in other clinicopathological features. In cohort 2, there were 34 patients (31.8%) in the HER2-zero group and 73 patients (68.2%) in the HER2-low group. No statistically significant differences were observed in clinicopathological features between the two groups. In cohort 3, there were 70 patients (37.0%) in the HER2-zero group and 119 patients (63.0%) in the HER2-low group. Histological grades were worse in the HER2-zero than in the HER2-low group (*p* = 0.029). Axillary lymph node dissection (ALND) was undergone more frequently in the HER2-zero than in the HER2-low group (*p* = 0.043). Other clinicopathological features did not differ significantly between the two groups.

**Table 2:** Patients’ characteristics according to the risk cohort.

Category	Cohort 1					Cohort 2					Cohort 3				
	HER2-zero (n = 124)		HER2-low (n = 227)		p-value	HER2-zero (n = 34)		HER2-low (n = 73)		p-value	HER2-zero (n = 70)		HER2-low (n = 119)		p-value
	n	%	n	%		n	%	n	%		n	%	n	%	
Age, years															
< 65	92	(74.2)	173	(76.2)	0.674	26	(76.5)	63	(86.3)	0.206	57	(81.4)	89	(74.8)	0.293
≥ 65	32	(25.8)	54	(23.8)		8	(23.5)	10	(13.7)		13	(18.6)	30	(25.2)	
Sex															
Female	123	(99.2)	225	(99.1)	0.942	34	(100)	71	(97.3)	0.330	70	(100)	119	(100)	N. A
Male	1	(0.8)	2	(0.9)		0	(0)	2	(2.7)		0	(0)	0	(0)	
Menopausal status															
Pre	54	(43.6)	111	(48.9)	0.337	18	(52.9)	41	(56.2)	0.755	31	(44.3)	66	(55.5)	0.138
Post	70	(56.5)	116	(51.1)		16	(47.1)	32	(43.8)		39	(55.7)	53	(44.5)	
ER status															
Negative	2	(1.6)	3	(1.3)	0.826	0	(0)	0	(0)	N. A	0	(0)	0	(0)	N. A
Positive	122	(98.4)	224	(98.7)		34	(100)	73	(100)		70	(100)	119	(100)	
PR status															
Negative	17	(13.7)	25	(11.0)	0.457	2	(5.9)	6	(8.2)	0.669	3	(4.3)	4	(3.4)	0.745
Positive	107	(86.3)	202	(89.0)		32	(94.1)	67	(91.8)		67	(95.7)	115	(96.6)	
HER2 status															
IHC0	124	(100)	0	(0)	< 0.001	34	(100)	0	(0)	< 0.001	70	(100)	0	(0)	< 0.001
IHC1+	0	(0)	151	(66.5)		0	(0)	53	(72.6)		0	(0)	88	(74.0)	
IHC2+/FISH−	0	(0)	76	(33.5)		0	(0)	20	(27.4)		0	(0)	31	(26.1)	
Histological type															
IDC	95	(76.6)	186	(81.9)	0.204	27	(79.4)	66	(90.4)	0.110	58	(82.9)	102	(85.7)	0.721
ILC	22	(17.7)	23	(10.1)		4	(11.8)	1	(1.4)		10	(14.3)	12	(10.1)	
IDC + ILC	2	(1.6)	4	(1.8)		1	(2.9)	1	(1.4)		0	(0)	1	(0.8)	
Others	5	(4.0)	14	(6.2)		2	(5.9)	5	(6.9)		2	(2.9)	4	(3.4)	
HG															
1	9	(7.3)	19	(8.4)	0.933	3	(8.8)	8	(11.0)	0.735	20	(28.6)	53	(44.5)	0.029
2	46	(37.1)	84	(37.0)		31	(91.2)	65	(89.0)		50	(71.4)	66	(55.5)	
3	69	(55.7)	124	(54.6)		0	(0)	0	(0)		0	(0)	0	(0)	
Tumor size, cm															
< 2	27	(21.8)	59	(26.0)	0.190	12	(35.3)	37	(50.7)	0.137	39	(55.7)	67	(56.3)	0.937
2–5	54	(43.6)	110	(48.5)		22	(64.7)	36	(49.3)		31	(44.3)	52	(43.7)	
≥ 5	43	(34.7)	58	(25.6)		0	(0)	0	(0)		0	(0)	0	(0)	
Number of positive lymph nodes															
1–3	53	(42.7)	114	(50.2)	0.180	34	(100)	73	(100)	N. A	70	(100)	119	(100)	N. A
≥ 4	71	(57.3)	113	(49.8)		0	(0)	0	(0)		0	(0)	0	(0)	
Ki-67 index, %															
< 20	38	(30.7)	68	(30.0)	0.893	0	(0)	0	(0)	N. A	70	(100)	119	(100)	N. A
≥ 20	86	(69.4)	159	(70.0)		34	(100)	73	(100)		0	(0)	0	(0)	
Breast surgery															
BCS	38	(30.7)	56	(24.7)	0.227	17	(50.0)	25	(34.3)	0.120	29	(41.4)	54	(45.4)	0.597
TM	86	(69.4)	171	(75.3)		17	(50.0)	48	(65.8)		41	(58.6)	65	(54.6)	
Axillary surgery															
SLNB only	6	(4.8)	10	(4.4)	0.852	2	(5.9)	8	(11.0)	0.401	9	(12.9)	30	(25.2)	0.043
ALND	118	(95.2)	217	(95.6)		32	(94.1)	65	(89.0)		61	(87.1)	89	(74.8)	
RT															
Yes	110	(88.7)	168	(74.0)	0.001	17	(50.0)	32	(43.8)	0.551	32	(45.7)	50	(42.0)	0.620
No	14	(11.3)	59	(26.0)		17	(50.0)	41	(56.2)		38	(54.3)	69	(58.0)	
CT															
Neoadjuvant	20	(16.1)	52	(22.9)	0.310	3	(8.8)	7	(9.6)	0.939	1	(1.4)	1	(0.8)	0.677
Adjuvant	86	(69.4)	147	(64.8)		23	(67.7)	51	(69.9)		31	(44.3)	46	(38.7)	
No	18	(14.5)	28	(12.3)		8	(23.5)	15	(20.6)		38	(54.3)	72	(60.5)	
ET															
Yes	115	(92.7)	223	(98.2)	0.009	33	(97.1)	71	(97.3)	0.953	66	(94.3)	114	(95.8)	0.637
No	9	(7.3)	4	(1.8)		1	(2.9)	2	(2.7)		4	(5.7)	5	(4.2)	

*ER* estrogen receptor, *PR* progesterone receptor, *HER2* human epidermal growth factor receptor 2, *IDC* invasive ductal carcinoma, *ILC* invasive lobular carcinoma, *HG* histological grade, *BCS* breast-conserving surgery, *TM* total mastectomy, *SLNB* sentinel lymph node biopsy, *ALND* axillary lymph node dissection, *RT* radiotherapy, *CT* chemotherapy, *ET* endocrine therapy, *FISH* fluorescence in situ hybridization.

HER2-zero group: Patients with immunohistochemistry (IHC) 0 score.

HER2-low group: Patients with IHC1+ or IHC2+/FISH− scores.

Cohort 1: Patients with ≥ 4 positive axillary lymph nodes (ALNs), or 1–3 positive ALNs, and either histological grade (HG) 3 or tumor size ≥ 5 cm.

Cohort 2: Patients with 1–3 positive ALNs, HG < 3, tumor size < 5 cm, and high Ki-67 index (≥ 20%).

Cohort 3: Patients with 1–3 positive ALNs, HG < 3, tumor size < 5 cm, and low Ki-67 index (< 20%).

### Recurrence events

The recurrence events of all patients are presented in Table 3. The median follow-up time was 71.9 months (50.0–101.8 months). During the follow-up period, 111 IDFS events and 79 DRFS events occurred. Most IDFS events were distant recurrences in the whole cohort. Common sites of distant recurrence were bone, liver, and lung. The detailed recurrence events of each cohort are also shown in Table 3.

**Table 3:** Recurrence events.

IDFS events	All patients (n = 647)	Cohort 1 (n = 351)	Cohort 2 (n = 107)	Cohort 3 (n = 189)
Total IDFS events	111	78	15	18
Patients with invasive disease, first occurrence	108	76	15	17
Local/regional recurrence	26	17	4	5
Distant recurrence	67	57	6	4
Contralateral recurrence	4	2	0	2
Second primary neoplasm	20	8	5	7
Death from any cause without invasive disease	3	2	0	1
DRFS events				
Total DRFS events	79	63	8	8
Patients with distant relapse, any time	69	59	6	4
Bone	40	34	2	4
Liver	19	17	1	1
Lung	15	12	2	1
Brain	3	3	0	0
Lymph node	16	16	0	0
Pleura	1	1	0	0
CNS	2	1	1	0
Other^a^	2	2	0	0
Death from any cause without distant recurrence	10	4	2	4

a: included two cases with stomach metastasis.

Abbreviations: IDFS, invasive disease-free survival; DRFS, distant relapse-free survival; CNS, central nervous system.

Cohort 1: Patients with ≥ 4 positive axillary lymph nodes (ALNs), or 1–3 positive ALNs, and either histological grade (HG) 3 or tumor size ≥ 5 cm.

Cohort 2: Patients with 1–3 positive ALNs, HG < 3, tumor size < 5 cm, and high Ki-67 index (≥ 20%).

Cohort 3: Patients with 1–3 positive ALNs, HG < 3, tumor size < 5 cm, and low Ki-67 index (< 20%).

### IDFS

**Figure 1:**
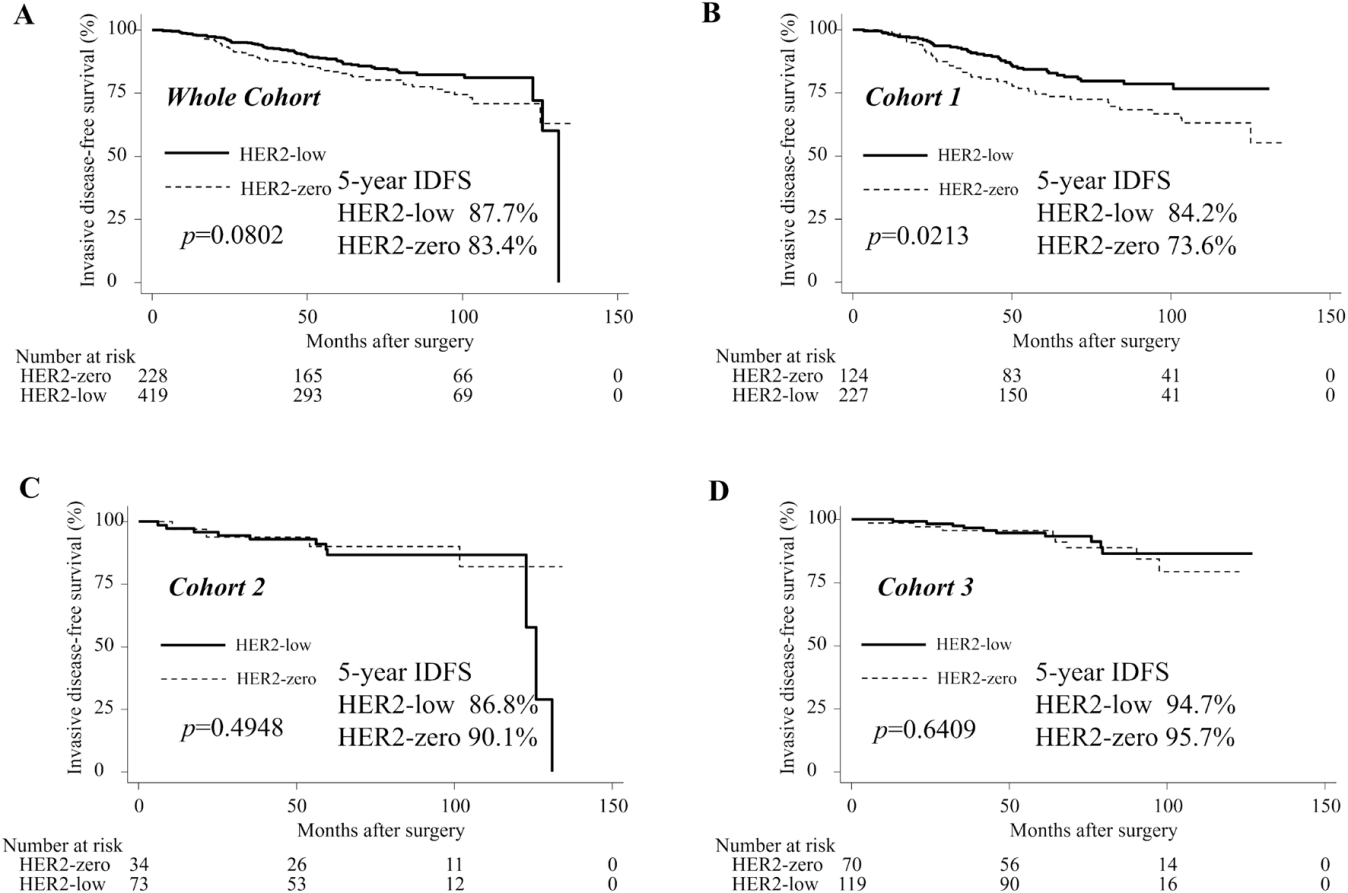
IDFS for the whole cohort and each cohort group. Kaplan–Meier curves of IDFS in HER2-low group and HER2-zero group are shown. 5-year IDFS and the p values for the log-rank test between the HER2-low group vs. the HER2-zero group are reported in each figure panel. (**A**) IDFS in Whole Cohort. (**B**) IDFS in Cohort 1. (**C**) IDFS in Cohort 2. (**D**) IDFS in Cohort 3. *IDFS* invasive disease-free survival, *HER2* human epidermal growth factor receptor 2. HER2-zero group: Patients with immunohistochemistry (IHC) 0 score. HER2-low group: Patients with IHC1+ or IHC2+/fluorescence in situ hybridization (FISH)—scores. Cohort 1: Patients with ≥ 4 positive axillary lymph nodes (ALNs), or 1–3 positive ALNs, and either histological grade (HG) 3 or tumor size ≥ 5 cm. Cohort 2: Patients with 1–3 positive ALNs, HG < 3, tumor size < 5 cm, and high Ki-67 index (≥ 20%). Cohort 3: Patients with 1–3 positive ALNs, HG < 3, tumor size < 5 cm, and low Ki-67 index (< 20%).

Kaplan–Meier survival estimates of IDFS for the whole cohort and each cohort group are shown in Fig. 1. In the whole cohort, there was no significant difference in 5-year IDFS between the HER2-zero and HER2-low groups (83.4% vs. 87.7%, *p* = 0.0802; Fig. 1A). However, in cohort 1, the HER2-zero group had a significantly poorer 5-year IDFS rate than the HER2-low group (73.6% vs. 84.2%, *p* = 0.0213; Fig. 1B). No significant differences were observed in 5-year IDFS between the HER2-zero and HER2-low groups in cohort 2 (90.1% vs. 86.8%, *p* = 0.4948; Fig. 1C) or 3 (95.7% vs. 94.7%, *p* = 0.6409; Fig. 1D).

### DRFS

**Figure 2:**
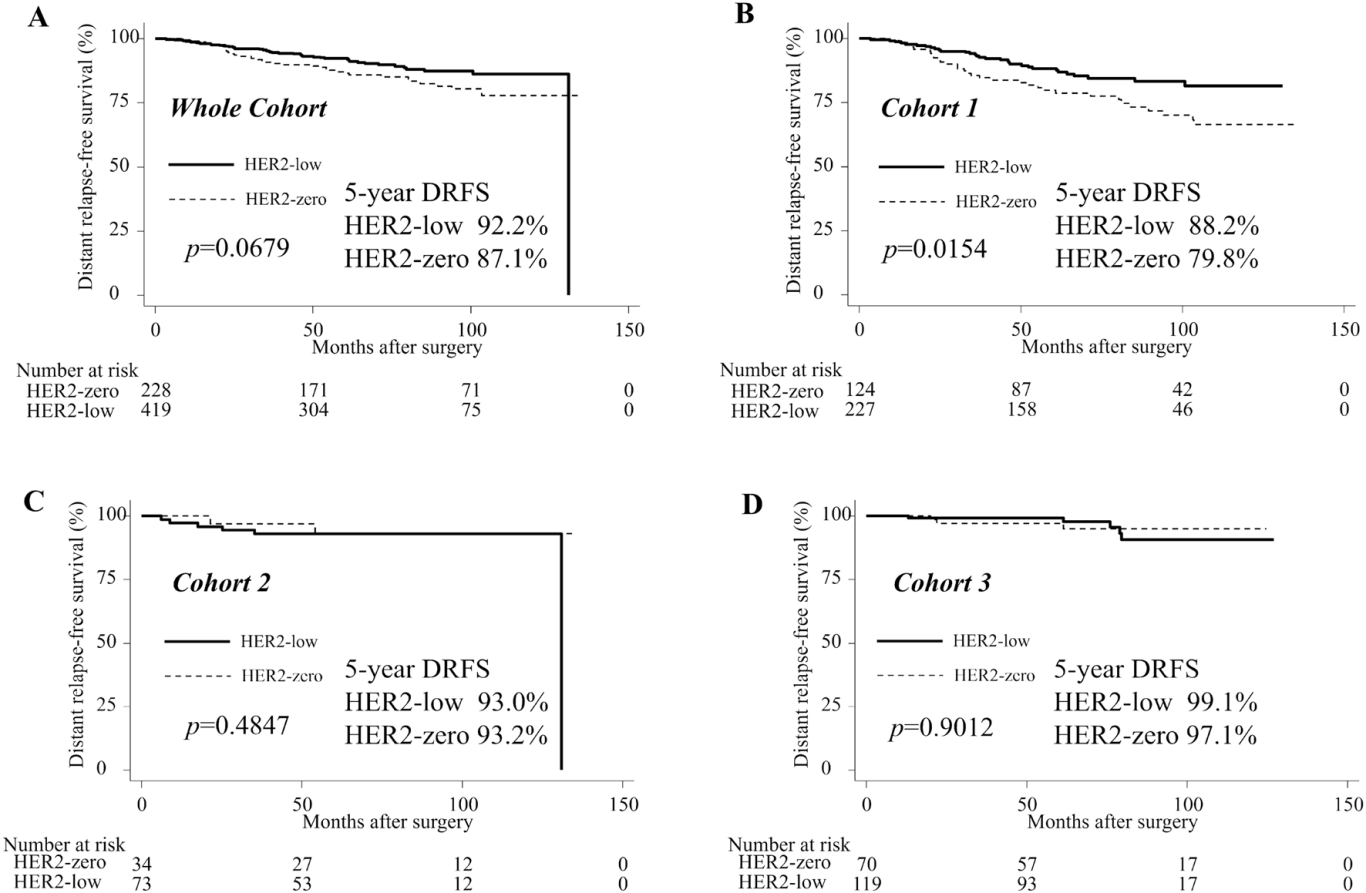
DRFS for the whole cohort and each cohort group. Kaplan–Meier curves of DRFS in HER2-low group and HER2-zero group are shown. 5-year DRFS and the p values for the log-rank test between the HER2-low group vs. the HER2-zero group are reported in each figure panel. (**A**) DRFS in Whole Cohort. (**B**) DRFS in Cohort 1. (**C**) DRFS in Cohort 2. (**D**) DRFS in Cohort 3. *DRFS* distant relapse-free survival, *HER2* human epidermal growth factor receptor 2. HER2-zero group: Patients with immunohistochemistry (IHC) 0 score. HER2-low group: Patients with IHC1+ or IHC2+/fluorescence in situ hybridization (FISH)—scores. Cohort 1: Patients with ≥ 4 positive axillary lymph nodes (ALNs), or 1–3 positive ALNs, and either histological grade (HG) 3 or tumor size ≥ 5 cm. Cohort 2: Patients with 1–3 positive ALNs, HG < 3, tumor size < 5 cm, and high Ki-67 index (≥ 20%). Cohort 3: Patients with 1–3 positive ALNs, HG < 3, tumor size < 5 cm, and low Ki-67 index (< 20%).

**Figure 3:**
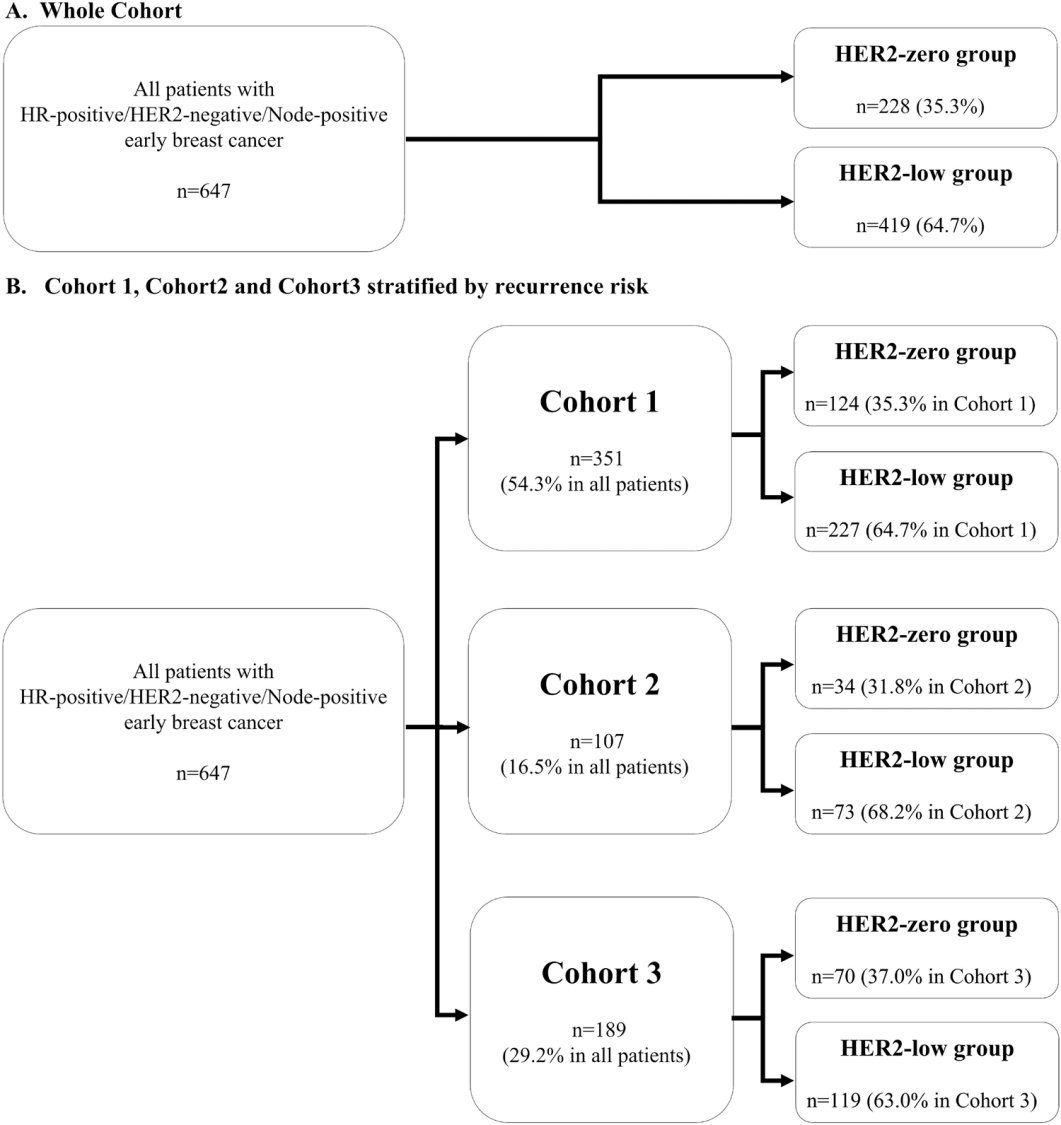
Study design. (**A**) Whole Cohort. (**B**) Cohort 1, Cohort 2 and Cohort 3 stratified by recurrence risk. *HR* hormone receptor, *HER2* human epidermal growth factor receptor 2. HER2-zero group: Patients with immunohistochemistry (IHC) 0 score. HER2-low group: Patients with IHC1+ or IHC2+/fluorescence in situ hybridization (FISH)—scores. Cohort 1: Patients with ≥ 4 positive axillary lymph nodes (ALNs), or 1–3 positive ALNs, and either histological grade (HG) 3 or tumor size ≥ 5 cm. Cohort 2: Patients with 1–3 positive ALNs, HG < 3, tumor size < 5 cm, and high Ki-67 index (≥ 20%). Cohort 3: Patients with 1-3 positive ALNs, HG < 3, tumor size < 5 cm, and low Ki-67 index (< 20%).

Kaplan–Meier survival estimates of DRFS for the whole cohort and each cohort group are displayed in Fig. 2. In the whole cohort, there was no significant difference in 5-year DRFS between the HER2-zero and HER2-low groups (87.1% vs. 92.2%, *p* = 0.0679; Fig. 2A). By contrast, the HER2-zero group had a significantly worse 5-year DRFS rate than the HER2-low group in cohort 1 (79.8% vs. 88.2%, *p* = 0.0154; Fig. 2B). No significant differences were seen between HER2-zero and HER2-low groups in cohort 2 (93.2% vs. 93.0%, *p* = 0.4847; Fig. 2C) or 3 (97.1% vs. 99.1%, *p* = 0.9012; Fig. 2D).

## Discussion

This study investigated the impact of HER2 expression level on the prognosis of patients with HR-positive, HER2-negative, node-positive EBC stratified by recurrence risk. The results revealed that patients with HER2-low had significantly more favorable IDFS and DRFS than patients with HER2-zero in the high recurrence-risk group (i.e., in cohort 1: patients with ≥ 4 positive ALNs, or 1–3 positive ALNs, and either HG 3 or tumor size ≥ 5 cm). By contrast, there were no significant differences in IDFS and DRFS between patients with HER2-low and HER2-zero in the low recurrence-risk group (i.e., in cohort 2: patients with 1–3 positive ALNs, HG < 3, tumor size < 5 cm, and high Ki-67 index (≥ 20%), and in cohort 3: patients with 1–3 positive ALNs, HG < 3, tumor size < 5 cm, and low Ki-67 index (< 20%)). These findings have not been reported previously.

There have been several reports regarding clinicopathological and prognostic differences between HER2-zero and HER2-low expression in patients with HR-positive, and HER2-negative EBC. Within HR-positive breast cancer, gene expression profiles were reportedly different between HER2-zero and HER2-low tumors^9^. HER2-zero tumors had a higher relative expression of proliferation-related genes and lower expression of luminal-related genes compared to HER2-low tumors. Luminal B and basal-like subtypes were more frequent in HER2-zero compared to HER2-low tumors (luminal B: 34.9% vs. 8.0%; basal-like: 33.4% vs. 1.9%), while luminal A subtype was more frequent in HER2-low compared to HER2-zero tumors (58.9% vs. 51.8%). Nevertheless, previous studies have presented conflicting results about whether there are prognostic differences between patients with HR-positive/HER2-low and HR-positive/HER2-zero EBC. Tan et al. have reported that patients with HER2-low non-metastatic breast cancer had favorable relapse-free survival (RFS) and OS compared to patients with HER2-zero breast cancer in both hormone receptor-positive and negative groups, but the absolute differences were modest and not clinically significant enough to support de-escalation of treatment^11^. Other studies have reported that no significant difference in prognosis between HER2-zero and HER2-low tumors was shown in patients with HR-positive, and HER2-negative EBC^9, 10^. In another study of HR-positive, HER2-negative EBC, HER2-low patients treated with neoadjuvant chemotherapy had significantly lower pathological complete response rate than those with HER2-zero, but no significant difference in DFS or OS were seen according to HER2 status^8^. In contrast to these studies, our study found significant differences in IDFS and DRFS between HER2-zero and HER2-low patients with a high risk of recurrence (5-year IDFS: 73.6% vs. 84.2%, *p* = 0.0213; 5-year DRFS: 79.8% vs. 88.2%, *p* = 0.0154, respectively). One possible reason for the differences between the results of the previous studies and our own could be differences in patient background. In our study, all patients were node-positive, whereas in previous studies, approximately 60% of the patients were node-negative and had a low risk of recurrence^8–10^. Interestingly, in patients with node-positive disease but who were considered at low risk of recurrence (i.e., cohort 2 and 3 in our study), there was no difference in prognosis according to HER2 expression. This trend was similar when RFS was evaluated as an endpoint. Patients with HER2-zero had significantly worse RFS than those with HER2-low in cohort 1, but not in cohort 2 or 3 (Supplemental Fig. 1). Similarly to our results, Mutai et al. have reported that the prognostic impact of HER2 expression varies depending on the risk of recurrence^12^. They reported the prognostic impact of HER2-low expression in HR-positive EBC differs between Oncotype DX risk groups. There was no significant difference in OS, DFS, or distant disease-free survival (DDFS) between patients with HER2-zero or HER2-low in the low recurrence score group (RS ≤ 25). Meanwhile, patients with HER2-zero had significantly worse OS, DFS, and DDFS than those with HER2-low in the high recurrence score group (RS > 25)^12^. These results suggest that the utility of HER2 expression as a prognostic factor in patients with HR-positive, HER2-negative, and node-positive breast cancer may differ depending on the risk of recurrence. In our present study, only 10 of the patients had the Oncotype DX test, making it impractical to assess the impact of risk classification based on the recurrence score. This limitation stemmed from the high cost associated with the Oncotype DX test and the results of the RxPONDER trial was not yet available as an option when patients in this study were making decisions regarding postoperative therapy. Consequently, there were limited opportunities to recommend or conduct the test.

In contrast, in our study, there was no significant difference in OS between patients with HER2-zero and patients with HER2-low, even in the high-recurrence risk group (Supplemental Fig. 2). Differences in the type of recurrence (distant vs. locoregional, visceral vs. non-visceral, etc.), deaths unrelated to breast cancer, shorter follow-up period, and fewer death events (total of 24 deaths) occurring in our study may have affected OS. Further evaluation of OS after a longer follow-up is required.

In our study, a significantly higher proportion of patients in the HER2-zero group than in the HER2-low group received RT in cohort 1 (88.7% vs. 74.0%, *p* = 0.001), and a significantly higher proportion of patients did not receive endocrine therapy (7.3% vs. 1.8%, *p* = 0.009). Regarding the disparity in RT rates in Cohort 1, differences were observed in the rates of PMRT for patients with 1–3 lymph nodes involved between the HER2-zero group (16 out of 28, 57.1%) and the HER2-low group (29 out of 80, 36.3%). Both groups exhibited a higher propensity for PMRT in cases where the tumor diameter exceeded 5 cm, while tending to forgo PMRT when the tumor diameter was less than 5 cm. Specifically, for patients with tumors larger than 5 cm in diameter, the PMRT rates were notably high, reaching 92.3% (12 out of 13) in the HER2-zero group and 93.8% (15 out of 16) in the HER2-low group. Conversely, for patients with tumors measuring 5 cm or smaller in diameter, the PMRT rate was 26.7% (4 out of 15) in the HER2-zero group and 15.6% (14 out of 64) in the HER2-low group. Notably, the HER2-low group had a higher proportion of patients with tumors measuring 5 cm or smaller in diameter, which may explain the lower PMRT rate observed in this group. Regarding the disparity in ET rates in Cohort 1, among the 9 patients in the HER2-zero group who did not receive ET, 3 patients did not undergo ET due to ER-negative/PR-positive tumors, 4 patients due to low ER levels, and 2 patients due to refusal of treatment. In the HER2-low group, 2 patients did not receive ET due to refusal of treatment. Among the 4 patients in the HER2-low group who did not receive ET, 1 patient did not undergo ET due to ER-negative/PR-positive tumors, 1 patient due to low ER levels, and 2 patients discontinued the medication one month after starting ET due to adverse events. It is noteworthy that the HER2-zero group had a higher number of patients who did not undergo ET due to ER-negativity or low ER levels compared to the HER2-low group. This difference in patient characteristics may have contributed to the observed variations in ET rates. Nevertheless, except for these points, the clinicopathological characteristics and treatment of the HER2-zero and HER2-low groups in each cohort were generally well-balanced (Table 2). Likely, the poorer prognosis of the HER2-zero compared to the HER2-low group in cohort 1 may have been influenced by the higher proportion of patients who did not receive ET. In fact, only nine patients in the HER2-zero group in cohort 1 did not receive ET, but four of them developed recurrences. Of these four patients, two refused systemic therapy at their own discretion, and the other two were ER-low positive (IHC < 10%) and received CT but not ET. Patients with ER-low-positive breast cancer have been reported to have significantly worse DFS and OS compared to patients with ER-positive breast cancer^13^. Although ER-low positivity may have affected prognosis in our study, we were not able to evaluate this point in detail because of the small number of ER-low-positive patients (11 in total). Although the number of patients who did not received ET and those who received only ET were limited due to the small number of patients in cohort 1 (13 and 44, respectively), we conducted additional prognostic analyses based on treatment modality. In cohort 1, which had only 13 patients without ET, marginal but not statistically significant differences were observed in 5-year invasive disease-free survival (IDFS) between the HER2-zero and HER2-low groups (53.3% vs. 100%, p = 0.0777; Supplementary Fig. 3A). However, among the 44 patients who received ET alone in cohort 1, the HER2-zero group exhibited a significantly poorer 5-year IDFS rate compared to the HER2-low group (56.1% vs. 91.0%, p = 0.0223; Supplementary Fig. 3B). Although some patients in cohort 1 received both chemotherapy (CT) and ET, there were no significant differences in 5-year IDFS between the HER2-zero and HER2-low groups (78.5% vs. 83.1%, p = 0.2261; Supplementary Fig. 3C). Nevertheless, these IDFS rates tended to be consistently lower in the HER2-zero group than in the HER2-low group during the follow-up period. Further evaluation of this point is warranted through long-term follow-up. Similar trends were also observed for distant relapse-free survival (DRFS) and recurrence-free survival (RFS) as for IDFS (Supplementary Figs. 4 and 5).

In our study, we opted for the risk classification of the monarchE trial over that of the PALLAS trial^14^ and the NATALEE trial^15^, which are analogous trials involving adjuvant CDK 4/6 inhibitors for high-risk HR-positive, HER2-negative EBC. We made this choice because the risk classification of the monarchE trial exclusively encompassed node-positive patients. Previous studies^9, 10^ did not reveal any significant prognostic differences between HER2-low and HER2-zero in HR-positive/HER2-negative EBC. This lack of significant findings was attributed to the inclusion of a substantial number of node-negative patients in these prior reports, who generally had a more favorable prognosis compared to node-positive patients. Consequently, we decided to limit our analysis to node-positive patients and considered the risk stratification of the monarchE trial to be most suitable for our study. The selection criteria of the PALLAS trial and NATALEE trial, which defined the high-risk population based on anatomical stage II or stage III, encompassed node-negative patients as well. We believed that these selection criteria did not align with the focus of our study. To compare the prognosis of HER2-low and HER2-zero while using the high-risk selection criteria of the NATALEE trial and the PALLAS trial, an analysis involving another population, including node-negative patients, would be necessary in the future. Additionally, we did not adopt the selection criteria of the OlympiA trial^16^, which included patients with germline pathogenic variants in BRCA1/2 EBC, mainly due to the absence of information regarding whether the patients in our study had germline pathogenic variants in BRCA1/2 mutations.

In the present study, the interpretation of HER2 was performed according to the ASCO/CAP guidelines. The first guideline on HER2 interpretation in breast cancer was provided in 2007. This was updated in 2013 and 2018, resulting in changes in the HER2 positive, equivocal, and negative criteria, mainly for HER2-IHC2+ cases^6, 17, 18^. During the study period, among 127 patients with HER2-IHC2+/FISH with no amplification, 116 (91.3%) patients were evaluated according to the ASCO/CAP guidelines for HER2 testing of the 2013 edition and the final decision was confirmed in our institution by several well-experienced pathologists. Therefore, it can be assumed that intra-institutional variability was low.

Our study suggested that differences in HER2 expression may be a prognostic factor for patients with HR-positive, HER2-negative breast cancer at high risk of recurrence. Although the efficacy of CDK 4/6 inhibitors has already been demonstrated in patients with high-risk HR-positive, HER2-negative EBC, it has not been fully evaluated whether HER2-low expression affects the clinical efficacy of CDK 4/6 inhibitors in patients with these high-risk HR-positive, HER2-negative EBC. Indeed, even regarding the efficacy of CDK 4/6 inhibitors in HR-positive, and HER2-negative metastatic breast cancer, there are still only a few reports. Shao et al. studied 45 patients with HR-positive, and HER2-negative metastatic breast cancer and reported no significant difference in objective response rate, disease control rate, and RFS between the HER2-zero and HER2-low groups^19^. Moreover, it has not been fully investigated whether CDK 4/6 inhibitors or anti-HER2 agents are more effective in patients with high-risk HR-positive, HER2-low positive primary breast cancer. These points should be evaluated in future studies.

This study had several limitations. First, it was a retrospective study performed at a single institution. Second, the sample size was not large and the follow-up periods may be too short to detect late recurrence five years from initial surgery. Third, the assessment of HER2 status based on different editions of ASCO/CAP guidelines might affect the results. Since only a few cases in this study had HER2 status assessments based on the ASCO/CAP 2018 edition, future studies are needed to evaluate whether basing HER2 status assessments on the latest guidelines would affect the results.

Despite the above limitations, our study was the first to reveal that HER2 expression level is a prognostic factor in HR-positive, HER2-negative, node-positive, high-risk EBC, using criteria in the monarchE trial. Our findings may provide useful information for treatment strategies in these patients.

## Methods

A total of 647 patients with HR-positive, HER2-negative, and node-positive who were diagnosed with primary breast cancer at the National Cancer Center Hospital between January 2011 and December 2019 were identified. The exclusion criteria were: (1) ductal carcinoma in situ at primary breast cancer, (2) stage IV disease at initial diagnosis, (3) patients without involved axillary lymph node at primary breast cancer, (4) patients with unknown receptor status, histological grade (HG), and Ki-67 index. The medical records of the included patients were procured from our retrospectively generated database to obtain patient age at initial diagnosis, sex, tumor size, nodal status, HG, histological type, menopausal status, estrogen receptor (ER) status, progesterone receptor (PR) status, HER2 status, Ki-67 index, type of initial surgery, and nature of any chemotherapy, postoperative radiotherapy, and endocrine therapy received.

### Pathological assessment and definition of receptor status

HR-positive was defined as either ER- or PR-positive. ER and PR were considered positive if the immunohistochemistry (IHC) staining was positive in more than 1% of tumor cells. A HER2-negative result corresponded to a score of 0, 1+ on immunohistochemistry (IHC), or 2+ on IHC with no amplification by FISH^6, 17, 18^. The HER2-low group was defined as patients with IHC1+ or IHC2+/FISH− scores, whereas the HER2-zero group was defined as patients with IHC0 scores. The TNM staging of breast cancer was based on the 8th edition of the American Joint Committee on Cancer staging manual^20^.

### Definition of recurrence risk group

Considering the risk of recurrence according to clinicopathological features applied in the monarchE study^2^, we classified patients into one of three cohorts. Cohort 1 included patients with ≥ 4 positive axillary lymph nodes (ALNs), or 1–3 positive ALNs, and either HG 3 or tumor ≥ 5 cm. Cohort 2 included patients with 1–3 positive ALNs, HG < 3, tumor size < 5 cm, and Ki-67 index ≥ 20%. Cohort 3 included patients with 1–3 positive ALNs, HG < 3, tumor size < 5 cm, and Ki-67 index < 20%. The Ki-67 index was evaluated using the same method used in the monarchE study. That is, the Ki-67 index was measured in all untreated breast primary tumor samples using the Ki-67 IHC assay developed by Agilent Technologies (formerly Dako; Santa Clara, CA)^21^ (Fig. 3).

### Statistical analyses

First, we compared the prognoses of the HER2-low and HER2-zero groups in all patients. We then compared the prognoses of the HER2-low and HER2-zero groups in each cohort. The baseline characteristics were evaluated using the Mann–Whitney *U* or Chi-square test, as appropriate. Five-year IDFS and 5-year distant relapse-free survival (DRFS) between HER2-zero and HER2-low groups were estimated using the Kaplan–Meier method and survival estimates were compared using the log-rank test. IDFS was defined as the time from the date of initial surgery to the date of the first occurrence of ipsilateral invasive breast tumor recurrence, local/regional invasive breast cancer recurrence, distant recurrence, death due to any cause, contralateral invasive breast cancer, or second primary non-breast neoplasm. DRFS was defined as the time from the date of initial surgery to the date of distant recurrence or death from any cause, whichever occurred first. All statistical analyses were conducted using the statistical software, STATA SE version 16 (StataCorp LP, College Station, TX). The threshold for significance was set as p < 0.05.

### Ethical approval

All procedures performed in this study involving human participants were in accordance with the Declaration Helsinki, and the National Cancer Center Hospital Review Board and Ethical Committee approved the present study (approval no. 2017-278). A waiver of informed consent was given by the National Cancer Center Hospital Review Board and Ethical Committee due to retrospective nature.

### Consent to participate

The requirement for informed consent was waived due to the retrospective nature of the study.

### Supplementary Information

Supplementary Figure 1.

Supplementary Figure 2.

Supplementary Figure 3.

Supplementary Figure 4.

Supplementary Figure 5.

## Data Availability

The datasets analyzed during the current study are available from the corresponding author upon reasonable request.
