# Supplementary material for: Prognostic impact of HER2-low positivity in patients with HR-positive, HER2-negative, node-positive early breast cancer
Source: Sci Rep. 2023 Nov 11;13:19669. doi: 10.1038/s41598-023-47033-8 (PMC10640570; doi:10.1038/s41598-023-47033-8)
Supplement: Supplementary file 1 — Supplementary Figure 1. [file 41598_2023_47033_MOESM1_ESM.docx]

**Prognostic impact of HER2-low positivity in patients with HR-positive, HER2-negative, node-positive early breast cancer**

*Scientific Reports*

Shohei Shikata^1^, Takeshi Murata^1*^, Masayuki Yoshida^2^, Hiromi Hashiguchi^1^, Yukiko Yoshii^1^, Ayumi Ogawa^1^, Chikashi Watase^1^, Sho Shiino^1^, Hirokazu Sugino^2^, Kenjiro Jimbo^1^, Akiko Maeshima^2^, Eriko Iwamoto^1^, Shin Takayama^1^, Akihiko Suto^1^

Correspondence should be addressed to:

Takeshi Murata

Department of Breast Surgery, National Cancer Center Hospital, 5-1-1 Tsukiji, Chuo-ku, Tokyo 104-0045, Japan.

Telephone number: +81-3-3547-5201

Fax number:+81-3-3542-3815

E-mail: tamurata@ncc.go.jp

ORCID: 0000-0003-0942-7599


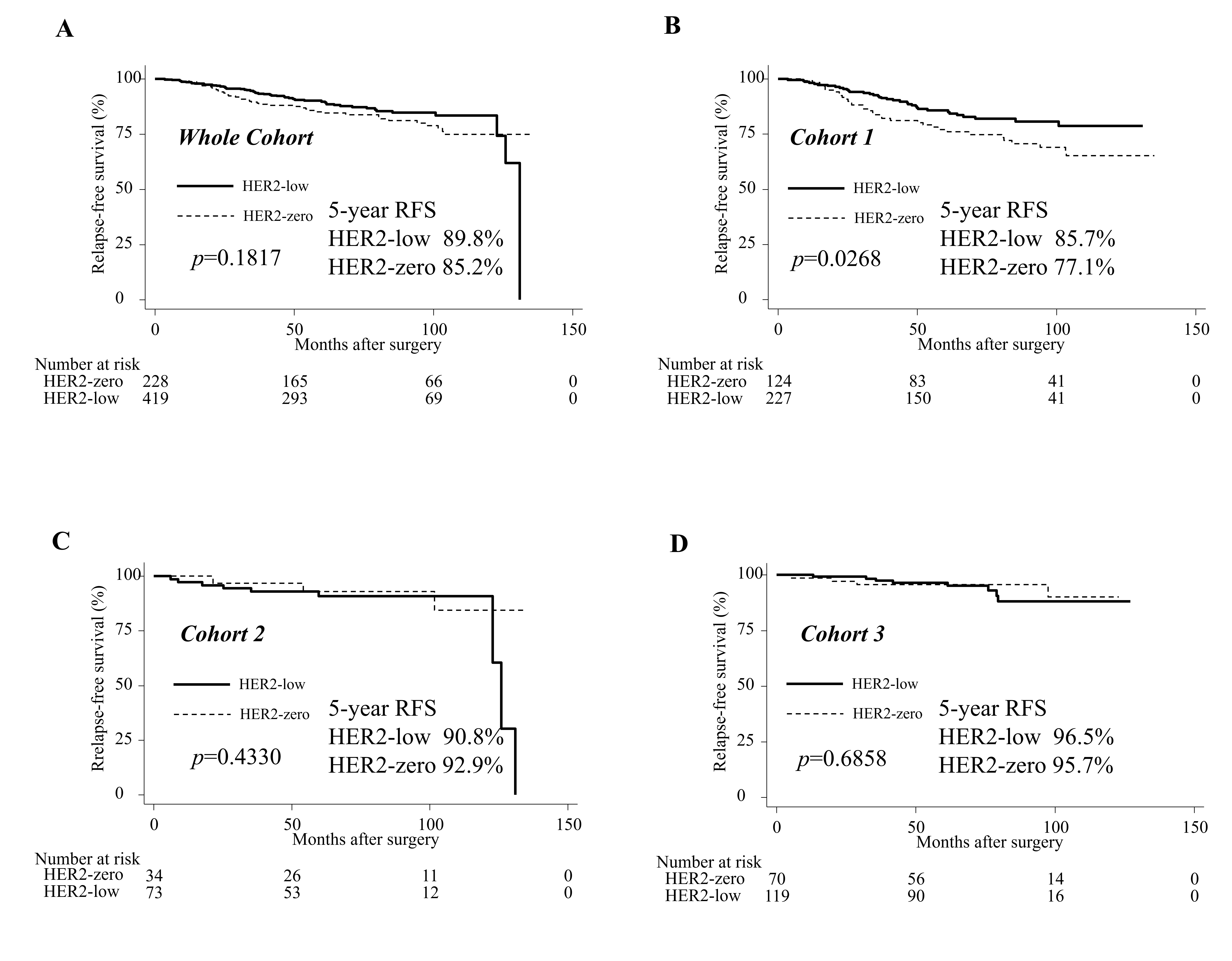


**Supplemental Figure 1. RFS for the whole cohort and each cohort group**

Kaplan-Meier curves of RFS in HER2-low group and HER2-zero group are shown. 5-year RFS and the *p* values for log-rank test between the HER2-low group vs HER2-zero group are reported in each Figure panel. **A** RFS in Whole Cohort. **B** RFS in Cohort 1. **C** RFS in Cohort 2. **D** RFS in Cohort 3.

Abbreviations: RFS, relapse-free survival; HER2, human epidermal growth factor receptor 2

HER2-zero group: Patients with immunohistochemistry (IHC) 0 score

HER2-low group: Patients with IHC1+ or IHC2+/fluorescence in situ hybridization (FISH) - scores

Cohort 1: Patients with ≥ 4 positive axillary lymph nodes (ALNs), or 1-3 positive ALNs, and either histological grade (HG) 3 or tumor size ≥ 5cm

Cohort 2: Patients with 1-3 positive ALNs, HG <3, tumor size < 5 cm, and high Ki-67 index (≥ 20%)

Cohort 3: Patients with 1-3 positive ALNs, HG <3, tumor size < 5 cm, and low Ki-67 index (< 20%)
